# Evaluation of a new patient consultation initiative in community pharmacy for ear, nose and throat and eye conditions

**DOI:** 10.1186/s12913-019-4125-y

**Published:** 2019-05-03

**Authors:** Gillian Hall, Tania Cork, Simon White, Hayley Berry, Louise Smith

**Affiliations:** 1 South Staffordshire Local Pharmaceutical Committee, Keele, UK; 2 Centre for Postgraduate Pharmacy Education, University of Manchester, Manchester, UK; 3 North Staffordshire and Stoke-On-Trent Local Pharmaceutical Committee, Keele, UK; 4 School of Pharmacy, Keele University, Keele, UK; 5 Faculty of Biology, Medicine and Health, University of Manchester, Manchester, UK

**Keywords:** Common ailments schemes, Minor ailments schemes, Self-care, Community pharmacy services

## Abstract

**Background:**

Community pharmacy Common Ailments Services can ease the considerable workload pressures on primary and secondary care services. However, evidence is needed to determine whether there are benefits of extending such services beyond their typically limited scope. This study therefore aimed to evaluate a new community pharmacy model of a service for patients with ear, nose and throat (ENT) and eye conditions who would otherwise have had to seek primary care appointments or emergency care.

**Methods:**

People with specified ENT or eye conditions registered with General Practitioners in Staffordshire or Shropshire who presented at participating community pharmacies were offered a consultation with a pharmacist trained to provide the service. The service included provision of relevant self-care advice and, where clinically appropriate, supply of non-prescription medicines or specified prescription-only medicines (POMs), including antibiotics, under Patient Group Directions. Patients received a follow up telephone call from the pharmacist five days later. Data were collected on the characteristics of patients accessing the service, the proportion of those who were treated by the pharmacist without subsequently seeing another health professional about the same condition, and patient reported satisfaction from a questionnaire survey.

**Results:**

A total of 408 patients accessed the service, of whom 61% received a POM, 15% received advice and medicine supplied under the common ailments service, 9% received advice and purchased a medicine, 10% received advice only and 5% were referred onwards. Sore throat accounted for 45% of diagnoses where a POM was supplied, 32% were diagnosed with acute otitis media and 15% were diagnosed with acute bacterial conjunctivitis. The number of patients successfully followed up was 309 (76%), of whom 264 (85%) had not seen another health professional for the same symptoms, whilst 45 (15%) had seen another health professional, usually their GP. The questionnaire was completed by 259 patients (response rate 63%) of whom 96% reported being very satisfied or satisfied with the service.

**Conclusions:**

The study demonstrates that pharmacists can effectively diagnose and treat these conditions, with a high degree of patient satisfaction. Wider adoption of such service models could substantially benefit primary care and emergency care services.

## Background

Common or Minor Ailments Services provided by community pharmacies offer an alternative location for patients to receive advice and treatment, rather than seeking treatment from their GP, out of hours provider (OOH), walk-in centre or accident and emergency department (A&E). This is part of an ongoing health policy move towards upskilling community pharmacists to provide more clinical services for patients and as a means of managing the considerable workload pressures on GPs, A&Es and OOHs [1–5]. In 2016–17 there were 23.4 million attendances at A&E in England, which compared to attendances in 2015–16 is an increase of 2% and an increase of 22% since 2007–08 [6]. Similarly, GPs’ overall workload was found to have increased by 16% between 2007 and 2014 [7], whilst a recent Kings’ Fund report found an increase in face-to-face patient contacts with GP surgeries of 2% and an increase of 24.5% in patient telephone contacts in 2016–17 compared to 2014–15 [8]. Subsequently, the GP Forward View promoted the provision of evidence-based common ailments services from community pharmacies [9].

The evidence to date suggests that when common ailments are managed by community pharmacists most patients’ symptoms resolve, few patients subsequently consult another health professional for the same condition and the cost of the pharmacist consultation is substantially lower than a consultation with a GP or attendance at A&E or OOH [10–23]. In a systematic review of 31 evaluations of minor ailment services, it was found that rates of symptom resolution ranged from 68 to 94% and reconsultation rates ranged from 2.4 to 23.4% [10]. A study published in 2015 compared the costs of consultations with community pharmacists with GP appointments and A&E attendances, and found the mean cost of community pharmacist consultations to be £29.30, which was nearly a third of the mean cost of GP appointments (£82.10) and nearly five times less than the mean cost of A&E attendances (£147,09) [11]. In a study that determined costs from where patients said they would have gone had the common ailment service not been available, it was estimated that the service resulted in a substantial reduction in local health care costs [12].

The range of common ailments eligible for management under common ailments services previously evaluated in the literature varies considerably, but does not usually include conditions for which antibiotics or other Prescription Only Medicines (POMs) may be supplied for, except in a minority of services [22]. These services only appear to have included infective conjunctivitis and uncomplicated urinary tract infection where antibiotics could be supplied [22]. The limited formulary that pharmacists may supply medicines from under common ailments schemes has been previously reported to be a barrier to patient care [23], which suggests that extension of such services to include common conditions requiring supply of antibiotics and other POMs may be beneficial.

However, an evaluation of an extended common ailments service that includes conditions requiring antibiotics or other POMs does not appear to have previously been reported in the literature and so we report an evaluation of such an extended service for common ear, nose and throat (ENT) conditions and bacterial conjunctivitis. The evaluation aimed to assess the outcomes of the extended service in terms of patient reported satisfaction, patient uptake characteristics and the proportion of patients accessing the service who could be treated by the pharmacist without them subsequently seeing another health professional about the same condition.

## Methods

### Description of the service initiative intervention

The Pharmacy First Extended Care Service for ENT & Eye conditions aimed to provide eligible patients registered with a GP practice contracted to NHS England, Staffordshire and Shropshire Area (NHSE S&S) with access to medication via community pharmacies for the treatment of specified ENT conditions and for acute bacterial conjunctivitis not suitable for treatment under the Pharmacy First Common Ailments Service (PFCAS). The specific ENT conditions included were acute otitis externa, acute otitis media, acute bacterial sinusitis, chronic sinusitis, seasonal allergic rhinitis, and sore throat. These ENT conditions were chosen on the basis of being among the most common category of presenting conditions to the local A&E departments and the specific conditions being commonly occurring within the category. Acute bacterial conjunctivitis was included because analysis of PFCAS data indicated that it was a common reason for GP referral by community pharmacists.

Consenting patients had a consultation with a pharmacist where their symptoms and their medical and medication history were assessed (with reference to their Summary Care Record wherever possible). The pharmacist examined the patient, assessed their treatment needs and determined whether they met specific inclusion criteria to be managed under the extended care service. For patients who were included, pharmacists gave self-care advice (including what to do if their symptoms did not resolve or their condition worsened) and, where clinically appropriate, supplied non-prescription medicines either purchased by the patient or through the PFCAS. Where antibiotic treatment or other POMs appeared to be necessary the pharmacist supplied these under Patient Group Directions (PGDs) specific to each condition and the GP practice was informed. The antibiotic that could be supplied was specified in each PGD (with an alternative in case of allergy or other contraindication) at a fixed dose, frequency and duration of treatment. Antibiotic choices in PGDs complied with local antibiotic formularies to take account of local antibiotic resistance patterns. The service also allowed deferring antibiotic treatment, which meant that if the patient returned after waiting a pre-defined number of days, the antibiotic could be supplied without repeating the consultation.

As part of accessing the service, all patients agreed to be contacted by the pharmacist approximately 5 days after their consultation for a short telephone interview to follow up on how successful the treatment had been and whether they had since seen another health professional for the same condition.

The service started in late November 2017 and was provided through ten community pharmacies contracted to NHSE S&S that had signed the Service Level Agreement (SLA). These pharmacies were selected for the service on the basis of having responded to a call for expressions of interest and meeting pre-defined criteria. These included that the pharmacies had to have an accredited consultation area approved for delivery of advanced services (i.e. clearly signed as a private consultation area, where the patient and pharmacist can sit down together and talk at normal speaking volume without being overheard by staff or customers) and that two pharmacists from each pharmacy had completed bespoke, online and face-to-face training on diagnosis and management of the ENT and eye conditions. The training was developed and delivered by a team that included two GP trainers in examination skills and pharmacist facilitators. This included training pharmacists on recognising ‘red flag’ signs and symptoms potentially indicative of a more serious condition, diagnosis in vulnerable groups and how to use diagnostic equipment (otoscope, torch and digital ear thermometer). Participating pharmacies were supplied with the additional equipment necessary (e.g. an otoscope) and were able to provide the service to a limited number of patients each, based on the funding secured. The service was funded by the North West Midlands Urgent and Emergency Care Network, which included the training and supply of additional equipment, and project managed by Local Pharmaceutical Committees (LPCs). Additional top-up funding was awarded to the project by NHSE S&S Local Professional Network for pharmacy.

### Data collection and analysis

The evaluation used two methods of data collection: *PharmOutcomes* to record service access data and a questionnaire survey as a means of collecting patient satisfaction data. *PharmOutcomes* was used as a means of collecting data about patient uptake and outcomes of the service because it is already used by community pharmacies in the study location to record and be remunerated for delivery of commissioned services. Recording modules can be written for this web-based system to match service specifications and PGDs, resources and links can be embedded into the system to allow the pharmacy team easy access to local documents and any relevant national guidance. Recording service delivery data on the system allows local and national level analysis on the effectiveness of commissioned services. All personally identifiable information was removed from the data downloaded from *PharmOutcomes* for the evaluation, but patient age, gender, ethnicity and the first part of their postcode were included. Anonymous patient satisfaction data were collected using a questionnaire because community pharmacy customers tend to be familiar with the format as it is commonly used by pharmacies and GP surgeries for gaining patient feedback. As a service evaluation, ethical approval was not required.

Questions were set up in *PharmOutcomes* for pharmacists to answer as consultations progressed to guide the consultation and ensure that standardised information was recorded. These questions were mapped to the SLA and PGDs. This included the outcome of pharmacists’ consultations with patients in terms of supply of non-prescription medicine (either purchased or supplied under the PFCAS), as well as the diagnosis made and the numbers of those who were supplied with a POM, not supplied with any treatment or were referred.

For the follow up telephone interviews, pharmacists attempted to contact all patients who had received the extended care service to determine whether they had subsequently seen another health professional about the same condition within 5 days of seeing the pharmacist and, if so, which professional they had seen. This data was recorded in *PharmOutcomes*. Where patients had seen another health professional they were asked why and a free text response was recorded in *PharmOutcomes*. This data was thematically categorised and frequency counted.

Patients who received the extended care service were also given a pack containing an invitation letter, a patient satisfaction questionnaire and a pre-paid envelope. They were invited to complete the questionnaire anonymously and post it back to the pharmacy, or anonymously complete an online version. The questions were designed as fixed response rather than free text and were developed from standard questions used in numerous other evaluations according to the aims of the evaluation of the extended care service. These included whether patients would use the service again, or recommend the service to others, reasons for using the service, and how they found out about the service, as well as asking them to rate their satisfaction with the service they had received on a 5 point Likert scale.

Data recorded in *PharmOutcomes* were downloaded into a Microsoft Excel spreadsheet and anonymised before being sent to a researcher (SW) who was independent of the service delivery and administration. Questionnaire data was manually inputted into Excel. These data were subjected to descriptive statistical analysis.

## Results

### Service access data

A total of 408 patients accessed the service, of whom 247 (60.5%) were women and 161 (39.5%) were men. Patients accessed the extended care service between late November 2017 and 6th March 2018, at which point the total allocated number of patients had been reached. The majority of patients (323, 79.2%) selected White British as their ethnicity, with other White ethnic origins selected by 11 (2.7%) of respondents and 18 (4.4%) respondents selecting Black, Asian or Minority Ethnic groups. A total of 53 (13%) selected the ‘prefer not to say’ option about ethnicity.

Table 1 shows the outcomes of consultations recorded in *PharmOutcomes*. The data indicates that for over 60% of patients this resulted in supply of a POM, whilst the remaining third of patients were managed with non-prescription medicines (also known as over the counter (OTC) medicines) and / or self-care advice, and only a small minority were referred for medical attention. The diagnoses made for those patients who were supplied with a POM are shown in Table 2, of which sore throat and acute otitis media were the most common. Table 3 shows the numbers and percentages of patients supplied with POMs for each indication and the numbers and percentages of deferred antibiotics.

**Table 1:** Patient outcomes from pharmacist consultations

Outcomes	No. (%)
POM supplied	249 (61)
Advice + OTC medicine supplied via PFCAS	60 (15)
Advice only (no medicines supplied)	41 (10)
Advice + OTC medicine purchased	38 (9)
Onward referral	20 (5)
Total	408 (100)

**Table 2:** Indications for POMs supplied

Outcomes	No. (%)
Sore throat	112 (45)
Acute otitis media	79 (32)
Acute bacterial conjunctivitis	36 (15)
Acute bacterial sinusitis	16 (6)
Acute otitis externa with suspected secondary infection	3 (1)
Chronic sinusitis	3 (1)
Total	249 (100)

**Table 3:** The number of patients supplied with POMs for each indication and the percentage of deferred antibiotics

Indication for POM supply	POM supplied	No. & % patients treated for that condition
Acute bacterial conjunctivitis	Chloramphenicol eye drops 0.5%	29 (80%)
	Chloramphenicol eye drops 0.5% (deferred)	1 (3%)
	Fusidic acid eye drops 1%	6 (17%)
	Fusidic acid eye drops 1% (deferred)	0
Acute otitis externa with suspected secondary infection	Clioquinol and flumetasone ear drops	3 (100%)
Acute otitis media	Amoxicillin	66 (84%)
	Amoxicillin (deferred)	9 (11%)
	Clarithromycin	4 (5%)
	Clarithromycin (deferred)	0
Acute bacterial sinusitis	Doxycycline	14 (88%)
	Doxycycline (deferred)	0
	Clarithromycin	2 (12%)
	Clarithromycin (deferred)	0
Chronic sinusitis and seasonal affective rhinitis	Beclometasone nasal spray 50mcg	3 (100%)
Sore throat	Phenoxymethylpenicillin	93 (83%)
	Phenoxymethylpenicillin (deferred)	5 (4%)
	Clarithromycin	14 (13%)
	Clarithromycin (deferred)	0

Of the total of 408 patients who accessed the extended care service, 309 (76%) were successfully contacted by telephone approximately 5 days after their initial consultation. Of these, 264 (85%) reported that they had not seen another health professional. The other 45 (15%) patients reported that they had seen another health professional, which in the majority of cases (78%) had been a GP, but 11% reported having returned to the pharmacy and a small minority had seen a practice nurse (2%) or attended an OOH (2%). None of the patients had gone to A&E. The reasons reported by patients for this are shown in Table 4.

**Table 4:** Reasons reported by patients for reconsultations

Reconsultation type	Reason	No.
For GP appointments	Symptoms did not resolve	11
	Was referred at initial pharmacist consultation	8
	Appointment already booked for another matter	7
	Developed more serious illness	4
	Wanted second opinion	2
	Needed sick note	1
For practice nurse appointment	Consulted for additional matter	1
For returning to the pharmacy	Symptoms did not resolve	3
For going to an out-of-hours service	Adverse reaction to antibiotic	1
Total (reason unknown for 7 patients)		38

### Patient questionnaire data

Questionnaire responses were received from 259 patients, which represents a response rate of 63%. There were nearly three times the number of female respondents (185, 73%) compared to males (65, 27%) and the mean age for women and men was 32 and 39 respectively. The age range for females was 0–77 and for 4–80 for males, indicating that parents or guardians completed the questionnaire on behalf of younger patients. Age or gender data were not recorded for 9 patients.

The vast majority of patients reported being very satisfied or satisfied with the service, with only 3 (1%) patients reporting being unsatisfied. The question was not answered by 7 patients.

In addition, 97% of respondents agreed that community pharmacies are appropriate places to provide the extended care service, 98% of respondents reported that they would use the service again and 99% of respondents reported that they would recommend the service to others. The reasons for using the service that were selected by respondents is shown in Table 5, of which not needing an appointment to access the service was the most frequently chosen.

**Table 5:** Reasons selected by patients for using the extended care service

Reasons	No.
No need for an appointment	207
Did not have to wait for GP appointment	169
Close to home	150
Convenient opening times	134
Convenient location (near work / shops)	77

In terms of how respondents had found out that the extended care service was being provided, 61% of respondents reported finding out from their GP surgery, 29% from a community pharmacy providing the service and 7% from word of mouth.

## Discussion

Key findings from this study include that the majority of eligible patients’ ENT or eye conditions could be treated by the pharmacist without them subsequently seeking care from their GP, or an OOH or A&E for the same condition and that patient-reported satisfaction with this extended common ailments service was extremely high. Nearly half of all patients who accessed the service presented with sore throat and approximately a third had acute otitis media. Over 60% of patients seen were supplied with an antibiotic or other POM. The small proportion (15%) of patients who reported seeing another health professional for the same condition after the consultation with the pharmacist usually saw their GP and if not because they had been referred to the GP by the pharmacist, then they most likely did so because their symptoms had not resolved, despite an initial course of antibiotics.

The high degree of patient satisfaction was evident in their overall satisfaction rating, but also in the very high proportion who indicated that they would use the service again and recommend it to friends and family. Being able to access the service without an appointment seemed to be a key factor for using it, but being aware that the service was available was also important and previous studies have shown low awareness among the general public about the availability of consultation-based services from community pharmacies [24]. The high proportion of patients (61%) who reported finding out about the service from their GP surgery suggests that surgeries were actively signposting patients to the service and that this may have been a major reason for its success.

This study adds to what is already known about community pharmacists being able to effectively undertake patient consultations and reduce workload pressures on GPs and A&Es. Previous studies of common ailments services have shown that despite variation in service quality [25], community pharmacists can effectively treat eligible conditions, with low reconsultation rates [10–12, 26] and high patient acceptability [27–29].

The Pharmacy First Extended Care Service for ENT and Eye conditions included infective or chronic conditions that have not been included in previous types of common ailment service. Exceptions to this have included bacterial conjunctivitis treated with chloramphenicol eye drops which has been included in a minority of services [22], and a feasibility study of community pharmacy screening and treating streptococcal sore throat with antibiotics has also been reported [26]. This significantly adds to the range of conditions that can be diagnosed and treated in community pharmacies.

Previous studies have shown that common ailments services cost less than GP appointments or A&E attendances [10–12, 16, 18]. It was beyond the scope of this study to undertake a full economic evaluation of the Pharmacy First Extended Care Service for ENT and Eye conditions, but the service did appear to reduce GP and A&E workload, and so savings were likely to have been made as the majority of patients would otherwise have had to see a GP or attend an OOH or A&E for antibiotic treatment.

The key strength of this study is that it reports on a substantially extended model of a common ailments service, which requires pharmacists to have advanced clinical examination skills, using diagnostic equipment such as otoscopes. This is an important step in the further development in the management of common ailments in community pharmacy, especially as the conditions covered in the service were broadly included in a list of ailments appropriate for management in community pharmacy recently developed using consensus methodology, which supports the rationale for their inclusion [30]. Developments of this nature are all the more important as many areas in England have now decommissioned their traditional common ailments services following publication of recent guidance from NHS England on OTC products that should not be routinely prescribed in primary care [31]. This guidance includes products used for minor ailments and applies to patients who are exempt from paying prescription charges who may no longer be able to be supplied with these products under common ailments services unless their minor ailment meets specific criteria.

A weakness of the study is that the majority of patients who accessed the service reported themselves to be White British and so it is not known whether similar results would be found in a more diverse patient population. Other limiting factors include that the pharmacists had to express interest in order to participate and so may have been more motivated to provide the service than may be found if the service were more widely rolled out. The service requires appropriately trained pharmacists and participating pharmacies to have appropriate facilities, including appropriate diagnostic equipment, which has a cost implication for wider roll out. Successful implementation also requires patients to be aware that the service is available, but the findings of this study suggest that this can be effectively done by GP surgeries actively signposting patients to it.

However, whilst the data presented here suggest benefit for patients, as well as workload pressure reduction for GPs, evaluating the full benefit for patients and the health economy of any wider uptake of the service would require an appropriately designed clinical trial, with economic evaluation. A feasibility study would likely be needed first to refine the conditions to include in the service and identify viable primary and secondary outcomes to determine the effectiveness of the service. The economic evaluation should include clinical and humanistic outcomes in addition to economic outcomes [32]. Qualitative exploration of community pharmacists’ and GPs’ perspectives on the quality of the service provided might also yield useful insights.

## Conclusions

The study demonstrates that pharmacists can effectively treat these specific ENT and eye conditions, with a high degree of patient satisfaction and a low proportion of patients subsequently seeing another health professional for the same condition. This extends what is already known about community pharmacists being able to effectively undertake patient consultations and reduce workload pressures on GPs and A&Es. Wider uptake of this service model would be expected to be beneficial for patients and the health economy, but this would need to be confirmed by an appropriately designed clinical trial with economic evaluation. This may represent a natural evolution for community pharmacy common ailment schemes and further integrate pharmacy into multidisciplinary primary healthcare provision.

## Abbreviations

A&E: Accident and Emergency Department
ENT: Ear, nose and throat
GP: General (medical) practitioner
LPC: Local Pharmaceutical Committee
NHSE S&S: National Health Service England, Staffordshire and Shropshire Area
OOH: Out-of-Hours (medical service)
PFCAS: Pharmacy first common ailments scheme
PGD: Patient Group Direction
POM: Prescription only medicine
SLA: Service Level Agreement
